# Assessing the reliability of ChatGPT: a content analysis of self-generated and self-answered questions on clear aligners, TADs and digital imaging

**DOI:** 10.1590/2177-6709.28.5.e2323183.oar

**Published:** 2023-11-03

**Authors:** Orlando Motohiro TANAKA, Gil Guilherme GASPARELLO, Giovani Ceron HARTMANN, Fernando Augusto CASAGRANDE, Matheus Melo PITHON

**Affiliations:** 1 Pontifícia Universidade Católica do Paraná, Orthodontics (Curitiba/PR, Brazil).; 2 Southwest Bahia State University , Orthodontics (Jequié/BH, Brazil).

**Keywords:** ChatGPT, Artificial intelligence, Clear aligner, Temporary anchorage device, Digital image

## Abstract

**Introduction:**

Artificial Intelligence (AI) is a tool that is already part of our reality, and this is an opportunity to understand how it can be useful in interacting with patients and providing valuable information about orthodontics.

**Objective:**

This study evaluated the accuracy of ChatGPT in providing accurate and quality information to answer questions on Clear aligners, Temporary anchorage devices and Digital imaging in orthodontics.

**Methods:**

forty-five questions and answers were generated by the ChatGPT 4.0, and analyzed separately by five orthodontists. The evaluators independently rated the quality of information provided on a Likert scale, in which higher scores indicated greater quality of information (1 = very poor; 2 = poor; 3 = acceptable; 4 = good; 5 = very good). The Kruskal-Wallis H test (*p*< 0.05) and *post-hoc* pairwise comparisons with the Bonferroni correction were performed.

**Results:**

From the 225 evaluations of the five different evaluators, 11 (4.9%) were considered as very poor, 4 (1.8%) as poor, and 15 (6.7%) as acceptable. The majority were considered as good [34 (15,1%)] and very good [161 (71.6%)]. Regarding evaluators’ scores, a slight agreement was perceived, with Fleiss’s Kappa equal to 0.004.

**Conclusions:**

ChatGPT has proven effective in providing quality answers related to clear aligners, temporary anchorage devices, and digital imaging within the context of interest of orthodontics.

## RESUMO

**Introdução:**

A Inteligência Artificial (IA) é uma ferramenta que já faz parte de nossa realidade, e esta é uma oportunidade de entendermos como ela pode ser útil na interação com os pacientes e no fornecimento de informações valiosas sobre Ortodontia.

**Objetivo:**

O objetivo deste estudo foi avaliar a precisão do ChatGPT em responder a perguntas sobre Alinhadores transparentes, Dispositivos de ancoragem temporária, e Imagens digitais em Ortodontia.

**Métodos:**

45 perguntas e respostas foram geradas pelo ChatGPT 4.0 e analisadas separadamente por cinco ortodontistas que, de forma independente, avaliaram a qualidade das informações fornecidas, usando uma escala de Likert, na qual pontuações mais altas indicavam uma maior qualidade das informações (1 = muito ruim; 2 = ruim; 3 = aceitável; 4 = bom; 5 = muito bom). Aplicou-se o teste H de Kruskal-Wallis (*p* < 0,05) e comparações pareadas *post-hoc* com correção de Bonferroni.

**Resultados:**

Das 225 avaliações dos cinco avaliadores diferentes, 11 (4,9%) foram consideradas como muito ruins, 4 (1,8%) como ruins, e 15 (6,7%) como aceitáveis. A maioria foi considerada boa [34 (15,1%)] ou muito boa [161 (71,6%)]. Com relação às pontuações dos avaliadores, percebeu-se uma leve concordância, com o Kappa de Fleiss igual a 0,004.

**Conclusões:**

O ChatGPT mostrou eficácia em fornecer respostas de qualidade para questões relacionadas a Alinhadores transparentes, Dispositivos de ancoragem temporária e Imagens digitais.

## INTRODUCTION

Artificial Intelligence (AI) is the ability of digital computers or computer-controlled robots to perform tasks typically associated with intelligent beings,^1^ and has recently drawn much attention due to new developments in machine learning methods that incorporate multiple layers of artificial neural networks trained on big data^2^ or deep learning.^3^

According to its own description, ChatGPT is *“a large language model created by OpenAI. I am designed to understand and generate natural language text, and I have been trained on a massive amount of data to help answer questions and provide information on a wide variety of topics. My training data includes text from books, articles, websites, and other sources, and I am constantly learning and updating my knowledge base to improve my responses. I can assist with tasks such as language translation, summarization, question-answering, and more”* (https://chat.openai.com/chat).

Researchers have shown that ChatGPT can pass medical licensing tests and is useful in the peer review process.^4^ However, the excitement surrounding it has been matched by several ethical issues that could, and perhaps should, restrict its use.^5^

Regarding orthodontic treatment planning, different orthodontists may have distinct plans for a given situation. Before the start of the treatment process, careful treatment planning must be carried out ^6^. Treatment planning is an intricate process that relies heavily on the orthodontist’s subjective judgment, due to the thorough and deliberate evaluation of numerous variables.^7^ Studies have demonstrated that the level of agreement between orthodontists reviewing identical sets of case records is not very high.^8^ ^-^ ^10^

The use of AI for orthodontic diagnosis and treatment planning has shown good results,^11^ and automated systems have done it remarkably well, with accuracy and precision comparable to those of trained examiners,^12^ and the assisted software showed good agreement with AutoCEPH^©^ and manual tracing for all the cephalometric measurements.^13^

The accuracy, reliability, and content validity of information related to orthodontics topics provided by ChatGPT has not been previously evaluated. This is especially significant considering queries that might be posed by dental practitioners, orthodontists, and patients. Consequently, this study offers an in-depth content analysis of how ChatGPT handles providing information about results linked with the use of clear aligners, temporary anchorage devices and digital imaging in orthodontics.

## MATERIAL AND METHODS

A comprehensive content analysis was performed in a series of 45 questions related to three topics in orthodontics (Clear aligners, Temporary anchorage devices and digital imaging). The topics were considered on the basis of the most innovative in a survey of the Journal of Clinical Orthodontics website (*https://www.jco-online.com* < access July, 23th, 2023 >). 

It was asked for the chatGPT-4 (OpenAI, San Francisco, CA: OpenAI LP) the fifteen most frequent questions about 1 - Clear aligners, 2 - Temporary anchorage devices; 3 - Digital Imaging in orthodontics, and then it was asked to the AI to respond the same self-generated questions. 

All of th 45 questions and answers were obtained and saved (Table 1). These answers were analyzed by three researches with over 20 years of clinical and academic experience in orthodontics and two PhD students in orthodontics. The evaluators independently rated the quality of the answers provided on a Likert scale, in which higher scores indicated greater quality of information (1 = very poor; 2 = poor; 3 = acceptable; 4 = good; 5 = very good). Scoring was based on the combination of the best available scientific evidence and the clinical expertise. Before the scoring process began, a meeting was convened to establish a shared understanding of the scoring system among the evaluators.

**Table 1:** Questions and answers provided by the ChatGPT, and summary of evaluators scores.

Topics	n	Questions generated by ChatGPT 4.0	Answers generated by ChatGPT 4.0	Median	IQR	Range
Clear aligner	1	Effectiveness: Are clear aligners as effective as traditional braces in treating my orthodontic condition?	Clear aligners can be as effective as traditional braces for many types of orthodontic problems, including mild to moderate malocclusions, but severe or complex cases might still require traditional braces.	5.0	5.0	3.0-5.0
	2	Treatment duration: How long will I need to wear clear aligners?	The duration varies based on the individual's condition, but it typically ranges from 6 to 18 months. Some cases may take up to 2 years or more.	4.0	4.0	3.0-5.0
	3	Aligner visibility: Will people be able to notice that I'm wearing clear aligners?	Clear aligners are designed to be virtually invisible. Most people will not notice them unless they're looking closely.	4.0	4.0	1.0-5.0
	4	Pain or discomfort: Will clear aligners cause pain or discomfort?	Some discomfort or pressure is normal, especially when you first start using a new set of aligners. However, many people find clear aligners to be more comfortable than traditional braces.	4.0	4.0	4.0-5.0
	5	Maintenance and cleaning: How do I clean and maintain my clear aligners?	Clear aligners should be cleaned daily using a toothbrush and cold water. Hot water can warp the plastic. Special cleaning solutions are also available.	5.0	5.0	4.0-5.0
	6	Impact on daily life: Can I eat and drink while wearing clear aligners? Will they affect my speech?	Aligners should be removed when eating and drinking anything other than water. They can also cause temporary slight speech changes, but most people adapt quickly.	5.0	5.0	4.0-5.0
	7	Frequency of use: How many hours per day should I wear my aligners?	Clear aligners should be worn for 20 to 22 hours per day, and only removed for eating, drinking, brushing, and flossing.	5.0	5.0	4.0-5.0
	8	Follow-up appointments: How often will I need to see the orthodontist during my treatment?	Generally, you'll have a check-up every 6 to 8 weeks to monitor your progress and receive new sets of aligners.	2.0	2.0	1.0-4.0
	9	Aligner replacement: How often will I need to change my clear aligners?	Typically, you switch to a new set of aligners every one to two weeks, following your orthodontist's instructions.	5.0	5.0	4.0-5.0
	10	Treatment cost: How much does orthodontic treatment with clear aligners cost? Will insurance cover the treatment?	The cost varies based on the complexity of the case and geographic location. Insurance may cover part of the cost. Always check with your insurance provider and orthodontist for details.	3.0	3.0	1.0-5.0
	11	Dental hygiene during treatment: How will wearing clear aligners affect my oral hygiene routine?	Brush and floss after each meal before reinserting your aligners to maintain proper oral hygiene.	5.0	5.0	5.0
	12	Treatment suitability: Are there specific cases or conditions for which clear aligners are not suitable?	Clear aligners are suitable for many, but not all, orthodontic issues. Severely rotated teeth, large gaps, or certain types of malocclusions may require traditional braces.	5.0	5.0	5.0
	13	Post-treatment retainers: After treatment, will I need to wear a retainer to keep my teeth in their new position?	Yes, wearing a retainer post-treatment is crucial to prevent teeth from gradually shifting back to their initial positions.	5.0	5.0	5.0
	14	Potential side effects: Are there any side effects or risks associated with using clear aligners?	Some possible side effects include minor discomfort, speech alteration, dry mouth or excessive salivation. Rarely, an allergic reaction to the aligner material may occur.	5.0	5.0	4.0-5.0
	15	Emergency situations: What should I do if I lose or break my aligner before the replacement is due?	If an aligner breaks or gets lost, contact your orthodontist immediately. They may advise you to move to the next set or revert to the previous one until a replacement can be provided.	5.0	5.0	5.0
TADs	1	What are TADs?: How do TADs work in orthodontic treatment?	Temporary Anchorage Devices (TADs) are tiny titanium screws that are placed into the jawbone to act as stable anchors for moving teeth during orthodontic treatment.	5.0	5.0	4.0-5.0
	2	Procedure of placement: How are TADs placed in the mouth? Is it a surgical procedure?	TADs are placed using a minimally invasive procedure that can often be done under local anesthesia right in the orthodontist's office.	5.0	5.0	3.0-5.0
	3	Pain and discomfort: Does the placement of TADs hurt? Will there be pain or discomfort afterward?	Most patients report feeling pressure but not pain during TAD placement. Mild discomfort may occur for a day or so after placement.	5.0	5.0	4.0-5.0
	4	Effectiveness: How effective are TADs in treating my orthodontic condition?	TADs can be extremely effective in orthodontic treatment, allowing for more efficient and precise tooth movement that was previously difficult or impossible.	5.0	5.0	1.0-5.0
	5	Suitability: Are TADs suitable for everyone? Are there any age or health restrictions?	Most people are good candidates for TADs. However, conditions that affect bone quality may impact their success.	5.0	5.0	1.0-5.0
	6	Maintenance: How do I clean and maintain TADs?	TADs can be cleaned with a soft toothbrush and non-prescription mouth rinse. Your orthodontist will provide specific care instructions.	5.0	5.0	5.0
	7	Duration of treatment: How long will I need to have the TADs in my mouth?	The duration varies based on the specifics of each case, but TADs generally stay in place for a few months to over a year.	5.0	5.0	1.0-5.0
	8	Dietary changes: Will TADs affect what I can eat and drink?	There are no specific dietary restrictions, but you should avoid hard or sticky foods around the TAD area to prevent loosening.	5.0	5.0	4.0-5.0
	9	Oral hygiene: How do TADs impact my oral hygiene routine?	TADs don't significantly affect oral hygiene, but proper cleaning around the TAD is essential to prevent infection.	5.0	5.0	4.0-5.0
	10	Impact on daily life: Will TADs affect my speech or appearance?	TADs are relatively unobtrusive and do not significantly affect speech or appearance.	5.0	5.0	2.0-5.0
	11	Cost: How much does orthodontic treatment with TADs cost? Will insurance cover the treatment?	The cost varies depending on the treatment plan, geographic location, and insurance coverage. Always check with your insurance provider and orthodontist.	5.0	5.0	4.0-5.0
	12	Potential risks and complications: What are the risks and potential complications associated with TADs?	Risks include loosening of the TAD, infection, and irritation to the gums or mouth. However, serious complications are rare.	5.0	5.0	5.0
	13	Removal: Is the removal of TADs painful? What is the procedure like?	Removal is typically straightforward and can be performed with local anesthesia. It's generally less uncomfortable than the insertion.	5.0	5.0	3.0-5.0
	14	Activity limitations: Are there any activities to avoid while having TADs?	Most patients can carry on with their normal activities, but contact sports or other activities that could cause a direct blow to the mouth may require extra protection.	5.0	5.0	1.0-5.0
	15	Emergency situations: What should I do if a TAD becomes loose or falls out?	If a TAD becomes loose or falls out, contact your orthodontist immediately. They may replace it or adjust your treatment plan as necessary.	5.0	5.0	5.0-5.0
Digital imaging in orthodontics	1	What is digital imaging? How does it work in orthodontics?	Digital imaging is a technique that uses digital sensors instead of traditional photographic film to capture images. In orthodontics, it helps in precise diagnosis, planning, and treatment.	5.0	5.0	3.0-5.0
	2	Advantages: What are the benefits of digital imaging over traditional methods?	Digital imaging offers numerous advantages over traditional methods, including immediate image viewing, less radiation exposure, superior image quality, and easy image sharing and storage.	5.0	5.0	4.0-5.0
	3	Procedure: What is the process of taking digital images? Is it painful or uncomfortable?	The process is similar to taking traditional X-rays but quicker and more comfortable. A sensor is placed in the mouth, and the image is captured and instantly projected onto a computer screen.	3.0	3.0	1.0-5.0
	4	Safety: Is digital imaging safe? How much radiation is involved compared to traditional X-rays?	Digital imaging uses significantly less radiation than traditional film X-rays, making it safer for patients.	4.0	4.0	1.0-5.0
	5	Accuracy: How accurate are the images produced by digital imaging?	Digital images are highly accurate, showing minute details that can be overlooked in traditional imaging. They can be zoomed and enhanced for better diagnosis and treatment planning.	5.0	5.0	3.0-5.0
	6	Usage: How are these digital images used in my orthodontic treatment?	Digital images are used to diagnose orthodontic issues, plan treatments, monitor progress, and evaluate treatment outcomes.	5.0	5.0	5.0
	7	Security: How are my images stored and who has access to them?	Digital images are stored on secure servers and are typically backed up regularly. Access is restricted to your healthcare providers or others you grant permission to.	5.0	5.0	3.0-5.0
	8	Cost: Is digital imaging more expensive than traditional methods? Will my insurance cover it?	The cost can be slightly higher than traditional imaging due to the advanced technology. However, most insurance plans cover digital imaging as part of orthodontic treatment.	5.0	5.0	4.0-5.0
	9	Frequency: How often will I need to have these digital images taken?	The frequency of imaging depends on your specific orthodontic needs and treatment plan. Your orthodontist will guide you.	5.0	5.0	5.0
	10	Comparison with traditional methods: Can digital imaging detect more issues than traditional X-rays or photos?	Digital imaging can detect more issues and provide a more comprehensive view of your oral structures compared to traditional methods.	5.0	5.0	4.0-5.0
	11	Image availability: Will I be able to view my digital images?	Yes, you can typically view your digital images during your orthodontic visits. Some offices may also provide you access to your images through patient portals.	5.0	5.0	4.0-5.0
	12	Necessity: Is digital imaging necessary for my orthodontic treatment?	Digital imaging is not always necessary but can greatly assist in complex cases by offering precise and comprehensive visual information for planning and treatment.	5.0	4.0	4.0-5.0
	13	Children and digital imaging: Is this technology safe and appropriate for children?	Yes, digital imaging is safe and appropriate for children. In fact, the lower radiation exposure makes it an even better choice for pediatric patients.	4.0	4.0	3.0-5.0
	14	Allergies: Can I have a reaction or allergy to digital imaging?	There is no risk of allergic reactions with digital imaging as there is no physical material or substance involved that would trigger an allergy.	5.0	5.0	3.0-5.0
	15	Emergency situations: Can digital imaging help in emergency orthodontic situations?	Yes, digital imaging can provide immediate and detailed visual information that can assist in diagnosing and treating emergency orthodontic situations.	5.0	5.0	4.0-5.0

IQR = interquartile range.

The performed analysis was grounded in the principles of a crowd score strategy,^14^ given that the outcomes under examination (answers from ChatGPT) lack an established ‘ground truth’, and the evaluation of their quality is fundamentally subjective. We focused on the median scores given by the evaluators for each answer.^15^

## STATISTICAL ANALYSIS

The results of the evaluators scores were tabulated in Microsoft Excel software and analyzed in the Statistical Package for Social Sciences v. 25 (SPSS; SPSS Inc., Chicago, IL) program. For each question, the median, interquartile range (IQR), and full range of scores were determined. Evaluators were given a random identifier, and Fleiss’s kappa was utilized to evaluate the consistency in scores among them. The reliability of the questionnaire (comprising the questions) was gauged using Cronbach’s alfa. The Kruskal-Wallis H test was applied to discern differences in scores among the evaluators. All statistical analyses were performed with a significance level of *p*< 0.05, and when conducting *post-hoc* pairwise comparisons, the Bonferroni correction was applied to control for multiple testing.

## RESULTS

The questions and answers generated by the ChatGPT, and the median, interquartile interval and range of evaluators scores are presented in Table 1. In general, the evaluators rated ChatGPT as providing good information regarding the evaluated topics: Clear Aligners = 4.33 ± 1.189, TADs = 4.57 ± 1.015), Digital imaging = 4.49 ± 0.89. The median score for the three main topics was 5.0 for all, and did not show statistical difference among them (*p*> 0.05) (Table 1).

Table 2 presents descriptive data regarding clear aligners, TADs, and digital imaging. The clear aligner indicated more variability in the scores.

**Table 2:** Descriptive data regarding topics.

Topics	Mean	Standard deviation	Median	P value
Clear Aligners	4.33	1.189	5.0	0.356
TADs	4.57	1.015	5.0	
Digital Image	4.49	0.890	5.0	

Statistical difference for *p* < 0.05.

Table 3 presents the distribution of evaluators scores ranging from “very poor” to “very good.” The overall result showed that the highest percentage of scores in the total dataset was for the “Very Good” category (71.6%), followed by “Good” (15.1%) and “Acceptable” (6.7%). The “Poor” and “Very Poor” categories had the lowest percentages: 1.8% and 4.9%, respectively. 

**Table 3:** Overall number (n) and percentage (%) of median scores by each evaluator.

Evaluators		(1) Very Poor	(2) Poor	(3) Acceptable	(4) Good	(5) Very Good
1	n	1	0	4	6	34
	(%)	2.2%	0.0%	8.9%	13.3%	75.6%
2	n	2	2	6	12	23
	(%)	4.4%	4.4%	13.3%	26.7%	51.1%
3	n	8	1	1	3	32
	(%)	17.8%	2.2%	2.2%	6.7%	71.1%
4	n	0	0	1	4	40
	(%)	0.0%	0.0%	2.2%	8.9%	88.9%
5	n	0	1	3	9	32
	(%)	0.0%	2.2%	6.7%	20.0%	71.1%
Total	n	11	4	15	34	161
	(%)	4.9%	1.8%	6.7%	15.1%	71.6%

Statistical difference for *p* < 0.05.

These results suggest that the majority of evaluators have provided scores in the higher quality categories (“Good” and “Very Good”), indicating a generally positive assessment of the topics being evaluated. The low percentages in the “Poor” and “Very Poor” categories suggest that the quality of the topics was perceived to be quite satisfactory by the evaluators.

Regarding evaluators’ scores, a ‘slight agreement’ was perceived^16^, with a combined Fleiss’s Kappa of 0.004. The Kruskal-Wallis test indicated a significant variation in scores among the evaluators (*p* < 0.001). A detailed pairwise comparison of scores can be found in Table 4.

**Table 4:** Pairwise comparison of evaluators scores.

Evaluator	Bonferroni pairwise comparison	
	t	p
1 vs. 5	0	> 0.99
3 vs. 5	-1,899	0.061
1 vs. 4	-1,925	0.057
3 vs. 2	-0,155	0.877
1 vs. 2	2.149	0.034
2 vs. 5	-2,258	0.026
4 vs. 5	2.166	0.033
3 vs. 5	-0,018	0.061
1 vs. 3	1.844	0.069
4 vs. 2	0.042	> 0.001

Statistical difference for p<0.05.

## DISCUSSION

This article examined the contemporary applications of ChatGPT, an advanced AI, focusing on the accuracy and efficiency of the answers generated for questions on clear aligners, temporary anchorage devices and digital imaging in orthodontics. In the present study, the chatbot promptly generated answers to all the questions in a matter of seconds. These answers were then compared with those from experienced orthodontists. This study potentially stands as one of the pioneering cross-sectional assessments examining the precision of ChatGPT in addressing questions on contemporary orthodontics.

Even though AI is not a brand-new technology, ChatGPT has become popular and mainstream. Several AIs has been widely used in a variety of disciplines, most notably biological and medical diagnostics,^16^ and the data may be trained using clinical data sets and used for a variety of tasks in dental and medical diagnostics.^17^

Regarding AI used for diagnosis and treatment planning in orthodontics, one study reported that AI performed remarkably well, matching the trained examiners’ accuracy and precision.^12^ Another study showed good reliability when an AI analyzed cephalometric points and measurements.^18^ Another showed success rates of 93% for the diagnosis of extraction vs. nonextraction and 84% for the detailed diagnosis of extraction patterns.^19^ However, none of them referred to the field of orthodontics. As in this study the grand majority of the answers were considered as very good by the five evaluators, the answers that received the lowest scores were *“Q: Follow-up appointments: How often will I need to see the orthodontist during my treatment? / A: Generally, you’ll have a check-up every 6 to 8 weeks to monitor your progress and receive new sets of aligners.”* (median: 2.0); *“Q: Treatment cost: How much does orthodontic treatment with clear aligners cost? Will insurance cover the treatment? / A: The cost varies based on the complexity of the case and geographic location. Insurance may cover part of the cost. Always check with your insurance provider and orthodontist for details.”*(median: 3.0) and *“Q: Procedure: What is the process of taking digital images? Is it painful or uncomfortable? / A: The process is similar to taking traditional X-rays but quicker and more comfortable. A sensor is placed in the mouth, and the image is captured and instantly projected onto a computer screen.”* (median 3.0). The answers obtained through ChatGPT are blunt, and an orthodontist with poor training may interpret all the answers as true and may lead to some level of misinformation. However, a well-trained orthodontist can take advantage of their clinical experience and maximize the treatment with esthetics, function, and stability. In the case of only written text from ChatGPT, things become more complicated, since if we are now in a situation where the experts are not able to determine what’s true or not, we lose the middleman that we desperately need to guide us through complicated topics.^4^ Large linguistic algorithms excel in knowledge-based examinations, but often fall short when addressing medical or dental subjects and literature. For these AI-driven models to perform optimally, they necessitate training on high-caliber datasets. However, their current training on potentially biased datasets might explain the inaccuracies observed when responding to specific research-related inquiries.

Therefore, if AI is not a brand-new concept and idea, why has this new development become so mainstream? The concept of a textbot that writes extremely well about almost everything is interesting, and human curiosity may be the answer to this question. Even though ChatGPT gave good and strong answers about the three studied subjects, this is an AI that learns from a sizable text data set compiled from books, articles, and webpages. Orthodontics requires more precise answers. ChatGPT includes both science and false information found in advertisements, social media, and websites.

ChatGPT is an AI chatbot that condenses information, provides intelligent-sounding text, and appears to be plausible, but it needs to be more accurate in orthodontics.^20^ The results of this article were broad, realistic and comprehensive, demonstrating knowledge of the subject, but without delving into more specific details. However, because ChatGPT is not a search engine, the answer must also be inaccurate if the source information is inaccurate.^20^ While ChatGPT generally provides accurate answers about orthodontics, it faces limitations such as: (1) its inability to critically review or analyze findings from scientific literature, (2) a knowledge base that only extends up to 2021 and is not updated, (3) occasional misinterpretation of medical terminology, (4) an inability to distinguish between reputable and predatory journal sources, and (5) concerns regarding scientific precision, potential biases, and the risk of disseminating misinformation to users.^21^ ^,^ ^22^

By now, we should consider AI an auxiliary tool in the treatment plan, but one that does not substitute for the orthodontist. We can consider the use of ChatGPT inevitable. It is up to orthodontists to seek clinical and scientific evidence. Although there is a widespread interest in the use of ChatGPT, it was not extensively trained on biomedical data nor were its responses in particular. It is likely that patients and clinicians will turn to ChatGPT for assistance in interpreting laboratory data or understanding how to utilize clinical laboratory services.^1^ While ChatGPT is seen as a potentially beneficial tool in healthcare settings, concerns about its accuracy, reliability, and medicolegal implications persist.^15^ It is important not only to understand whether the use of AI tools is reliable in the clinical environment, but also to analyze their accuracy not just in one phase, but throughout the clinical workflow.^23^

ChatGPT’s orthodontics answers displayed an overall accuracy: out of 225 answers, the majority were rated as very good (71.6%) or good (15.1%). However, its effectiveness is limited by: an inability to critically analyze literature findings, a knowledge database restricted to 2021 (Version 4.0), incapability to distinguish between predatory and indexed journals, and a lack of scientific precision and reliability. In addition, the divergences in evaluator agreement and median scores highlight the possible inaccuracy in ChatGPT answers and the inherent subjectivity of outcomes. To mitigate this subjectivity, given the absence of an absolute standard, a crowd score approach was used based on earlier studies,^14^ involving multiple evaluators and aggregating median scores. This median score reflects evaluators’ collective agreement, while the interquartile range indicates divergence points. Despite evaluators’ varied expertise and anticipated diverse opinions, this diversity reflects the general consensus in orthodontics.^15^ Just as evaluators from distinct specialties naturally held varying views, their collective assessment reflects a consensus within orthodontics. 

ChatGPT has shown remarkable expertise in the assessed orthodontic topics, but differences in individual evaluations remind us of the complexity and subjectivity inherent in any assessment process. Patients and orthodontists need to be aware of the constraints and ethical issues surrounding ChatGPT, and should consistently verify information using reliable sources. Before incorporating these AI models into the healthcare system, efforts must be directed towards enhancing their reliability.

## CONCLUSION

The evaluation of ChatGPT’s knowledge and answers regarding the orthodontic topics of clear aligners, TADs and digital imaging suggests a generally favorable reception by the evaluators: with the majority of the scores grouped in the “Very Good” and “Good” categories, the data highlights ChatGPT’s ability to provide high-quality information in these areas. Median scores for all three main topics consistently reflected this positivity.
